# False Approximations of the Approximate Number System?

**DOI:** 10.1371/journal.pone.0025405

**Published:** 2011-10-12

**Authors:** Titia Gebuis, Maarten J. van der Smagt

**Affiliations:** 1 Experimental Psychology, Helmholtz Institute, Utrecht University, Utrecht, The Netherlands; 2 Laboratory of Experimental Psychology, KU Leuven, Leuven, Belgium; National Institute of Mental Health, United States of America

## Abstract

Prior research suggests that the acuity of the approximate number system (ANS) predicts future mathematical abilities. Modelling the development of the ANS might therefore allow monitoring of children's mathematical skills and instigate educational intervention if necessary. A major problem however, is that our knowledge of the development of the ANS is acquired using fundamentally different paradigms, namely *detection* in infants versus *discrimination* in children and adults. Here, we question whether such a comparison is justified, by testing the adult ANS with both a discrimination and a detection task. We show that adults perform markedly better in the discrimination compared to the detection task. Moreover, performance on discrimination but not detection, correlated with performance on mathematics. With a second similar experiment, in which the detection task was replaced by a same-different task, we show that the results of experiment 1 cannot be attributed to differences in chance level. As only task instruction differed, the discrimination and the detection task most likely reflect differences at the decisional level. Future studies intending to model the development of the ANS should therefore rely on data derived from a single paradigm for different age groups. The same-different task appears a viable candidate, due to its applicability across age groups.

## Introduction

The approximate number system (ANS) has been put forth as the foundation for our acquired mathematical abilities [1], [2]. A model describing the development of the ANS could therefore be a helpful tool to predict future mathematical abilities. However, researchers used fundamentally different paradigms to assess the ANS at different developmental stages. It is therefore unclear whether results from these different studies can be compared and incorporated into a single model, or whether a single paradigm for testing infants as well as children and adults might be more useful. In the current study we address the differences in the current paradigms and the impact this has on modelling the development of the ANS.

The ANS has been extensively studied in infants using the so-called *‘looking-time’ paradigm*. In studies employing this method, a stimulus (e.g. a random dot array) with the same numerosity content (often called a ‘standard’) is presented repeatedly, which results in a decrease in time spent looking at the stimulus (a phenomenon called ‘habituation’). The presentation of a stimulus with a distinct numerosity subsequently results in a looking-time increase. Such an increase in looking time can only be obtained if the infant is capable of detecting the number-deviant stimuli among the standards. This ‘response’ to changes in numerosity already increases in precision between the age of 6 to 10 months [3], [4], [5], [6]. The development of the ANS beyond infancy is mainly studied using the *numerosity discrimination task*. Here, children or adults perceive two random dot arrays and have to decide which of the two arrays represents more dots. The precision with which children can discriminate numerosities increases gradually with age [7] up to a ratio of around 7∶8 in adulthood [7], [8], [9], [10]. Interestingly, the precision with which children can differentiate numerosities has been shown to relate to their mathematical abilities earlier in life [1] and is dramatically impaired in dyscalculic children [10]. It is therefore suggested that the acuity of the ANS is fundamental to (future) mathematical abilities. Note, however, that other studies failed to replicate this finding, and instead revealed a relation between comparing symbolic number stimuli and math ability [11], [12].

In recent years, a number of studies emphasized the importance of generating models describing the development of the ANS from infancy to adulthood [1], [7], [10], [13]. Such models have the potential to detect deficits in the ANS at a very early age, which makes subsequent remediation more likely to be effective. However, before these models can be used as a tool to predict future mathematical abilities, the implicit assumption that the different paradigms used in different studies measure the ANS in a similar manner needs to be confirmed. In the two-alternative-forced-choice paradigms (administered to children and adults), where subjects judge which of two presented stimuli represents the larger numerosity, chance-level is at 50% (e.g. [7]). In contrast, numerosity discrimination ability is derived from *detection* paradigms in the infant studies, where subjects are expected to respond differently when a numerosity change is detected within a constant stream of (random-dot) images containing the same numerosity (e.g. [4], [5], [14]). Here, chance-level depends on the relatively small number of numerosity-change trials in relation to the numerosity-constant trials. The added uncertainty induced by this lower chance level could render this task much more difficult than the discrimination task. In addition, subjects could rely on different strategies to solve both tasks. Directly comparing the two paradigms within the same subjects is a necessity before any conclusions can be drawn about infant *and* child or adult data. Experiment 1 was designed to provide such a comparison.

## Experiment 1

In this first experiment we directly compared results derived from a numerosity detection and discrimination task. To only target the effect of task differences, stimulus properties in the two tasks were kept identical (see methods section). If both tasks measure approximate number processes in a similar manner, performance should be comparable. In addition, we analyzed performance on simple mathematical tasks (i.e. addition, subtraction, multiplication and division). Since the acuity of numerosity discrimination has been suggested to relate to mathematical abilities [1], [2], performance on the mathematical tasks should explain the variance in performance on this task. If both numerosity discrimination and detection measure the ANS in a similar manner, performance on the mathematical tasks should *also* explain variance in performance on the numerosity detection task.

### Methods

#### Participants

Twenty-six subjects participated in this study, of which twenty-four were included in the analyses (aged between 19 and 32 years; M = 23.3, SD = 3.53; 18 female, 6 male). Data from two subjects were discarded before analyses (the number of false alarms was more than 2 SD above average for one subject and the number of hits more than 2 SD below average for the other subject). All subjects were native Dutch speakers and had normal or corrected-to-normal vision. Written informed consent was obtained according to the Declaration of Helsinki and as approved by the local Ethical Committee.

#### Stimuli and procedure

In both the discrimination and detection task random dot patterns were presented in grey on a black background. The dot locations were randomized but constrained to an area of 7×7 degrees visual angle. The distance between dots was always at least 0.3 degrees visual angle. Individual dot sizes varied within the arrays between 0.4 and 0.8 degrees visual angle in diameter. The visual cues were controlled for in a similar manner in both tasks, for details see below. The order of the tasks was counterbalanced between subjects: half of the subjects started with the discrimination task while the other half started with the detection task.

*In the discrimination task,* each trial consisted of two random dot displays presented sequentially. For each trial, the first or the second display always represented twelve dots while the other display represented an equal, a smaller, or a larger number of dots. Five numerosities were included for trials representing a number smaller than twelve (ratio 2.0 (6 dots), ratio 1.5 (8 dots), ratio 1.33 (9 dots), ratio 1.2 (10 dots), ratio 1.09 (11 dots)) and larger than twelve (ratio 2.0 (24 dots), ratio 1.5 (18 dots), ratio 1.33 (16 dots), ratio 1.17 (14 dots) and ratio 1.08 (13 dots)). Each stimulus pair was presented 20 times (thus 11 ratio conditions x 20 trials, resulting a total of 220 trials). In half of the trials all the visual cues for dots of the more numerous display were larger when compared to the less numerous display presented in the same trial. For the other half of the trials, the reverse was true. In this manner, the average dot size, total surface area and the average contour length correlated positively with numerosity in only half of the trials, and negatively in the other half. To create these differences in visual properties, the dot sizes for the individual dots of each display were drawn from either a right- or left-skewed distribution containing all possible dot sizes. In other words, the chance that a small dot was drawn was increased in the right-skewed condition while the chance that a large dot was drawn was increased in the left-skewed condition. To create a number and visual cue correlated trial (the larger number consisted of larger visual parameters than the smaller number), the individual dots of the display of the larger number were drawn from the left-skewed distribution and the individual dots of the smaller number were drawn from the right-skewed distribution. For trials where number and visual parameters were anti-correlated (the larger number consisted of smaller visual parameters than the smaller number), the individual dots of the larger number were drawn from the right-skewed distribution and the individual dots of the smaller number from the left-skewed distribution. Note that a single dot size could be drawn multiple times (a comparable way to control for the visual cues of the stimuli is extensively described in [15]). The stimuli were presented for 300 ms with an inter-stimulus-interval of 800 ms.

The subjects were asked to indicate which of the two displays contained more dots, by pressing the corresponding button. In half of the trials the first contained more dots whereas in the other half of the trials the second display contained more dots. After a response was given, the next trial started. The task consisted of 2 blocks separated by a short break. Before the subjects started, they performed 15 practice trials, to familiarize themselves with the task.

**Figure 1:**
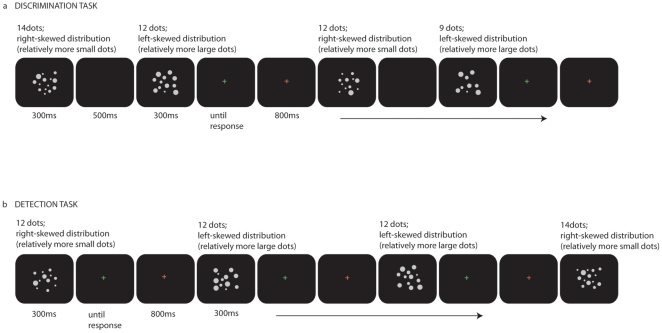
Stimulus examples of the discrimination (a) and the detection task (b).

In the *detection task*, subjects were presented with a continuous stream of displays of twelve dots, hereafter referred to as the baseline stimuli. Occasionally a display representing a larger or smaller number of dots was presented. These numerosity-deviant trials were always separated by four to eight baseline trials. The same 11 ratio conditions used in the discrimination task were incorporated in the detection task, and visual cues were controlled in an identical manner. For the numerosity-deviant trials, visual cues correlated positively with numerosity in half of the trials and negatively in the other half of the trials when compared to the baseline stimulus preceding it. To this end, dot sizes were again drawn from a left- or right-skewed distribution of possible dot sizes. In the remaining baseline trials, the sizes of the dots in a display were randomly drawn from the left-skewed or the right-skewed distribution. Thus in these baseline trials, the distribution from which the dot sizes were drawn did not necessarily alternate between sequentially presented trials (see Figure 1).

Before the task started, subjects were shown fifteen examples of the baseline. In this manner they could create a mental image of the baseline, without explicit information on the exact numerosity. During the task, subjects decided each trial whether it was a baseline or a numerosity-deviant trial by pressing the corresponding button. Stimuli were presented for 300 ms followed by a green cross, which turned red after the subject responded. The red cross remained on the screen for 800 ms. The task consisted of 5 blocks. Between blocks subjects could take a break. Each block always started with three examples of the baseline to refresh the subject's memory of what the baseline stimulus looked like. The task consisted of 11 different numerosity-deviant trials and between subsequent numerosity of (on average) 6 baseline trials. Each numerosity-deviant trial was presented 20 times. The task consisted of approximately 1500 trials.

For the *mathematical tasks*, subjects performed four different sets of mathematical problems (addition, subtraction, multiplication, division). Task difficulty increased with the number of problems solved and therefore subjects were stressed to solve the problems in the order presented. Increasing the number of digits in the mathematical problems and, in case of addition or subtraction, the requirement of carrying or borrowing resulted in the gradual increase in difficulty: addition (e.g. 2+5, 5+18, 26+13, 28+57), subtraction (e.g. 6-3, 17-4, 38-9, 82-38), multiplication (e.g. 3×9, 12×4, 53×5, 12×13) and division (e.g. 8/2, 81/9, 54/3, 85/5). These sets were presented to the subject separately and in a fully randomized order to overcome order effects. Subjects were instructed to solve as many problems within a set as fast and accurately as they could within one minute.

#### Analyses

For the discrimination task, the percentage of correct trials was calculated for each ratio condition. For the detection task, the number of hits per ratio condition and the total number of false alarms were calculated. We discarded subjects from the analyses that performed more than 2 SD above average for the number of false alarms or more than 2 SD below average for the number of hits (this resulted in the rejection of two subjects). First, we compared performance against chance level (discrimination 50% chance level; detection task 14% chance level as 1 out of 7 was a numerosity-deviant trial). P-values were adjusted for multiple comparisons using Bonferroni correction. Note that our current paradigm did not allow us to calculate the d-prime for each ratio condition in the detection task: we used different ratio conditions but only a single baseline condition. Hence the false alarms (incorrectly identifying a baseline condition as a target) could not be ascribed to a single target condition. However, only a negligible number of false alarms (3.4%) were made. Therefore, testing against chance level appears a valid measure of performance. Second, the effect of ratio, stimulus size and visual cues on task performance was investigated for each task separately using a repeated measures ANOVA. Third, we calculated percent correctly discriminated and percent correctly detected items across all ratio conditions, while for the mathematics tasks we calculated percent correctly solved problems for each task separately. A regression analyses was conducted to investigate whether performance on the mathematics tasks could explain the variance in performance on the discrimination and/or the detection task.

### Results

**Figure 2:**
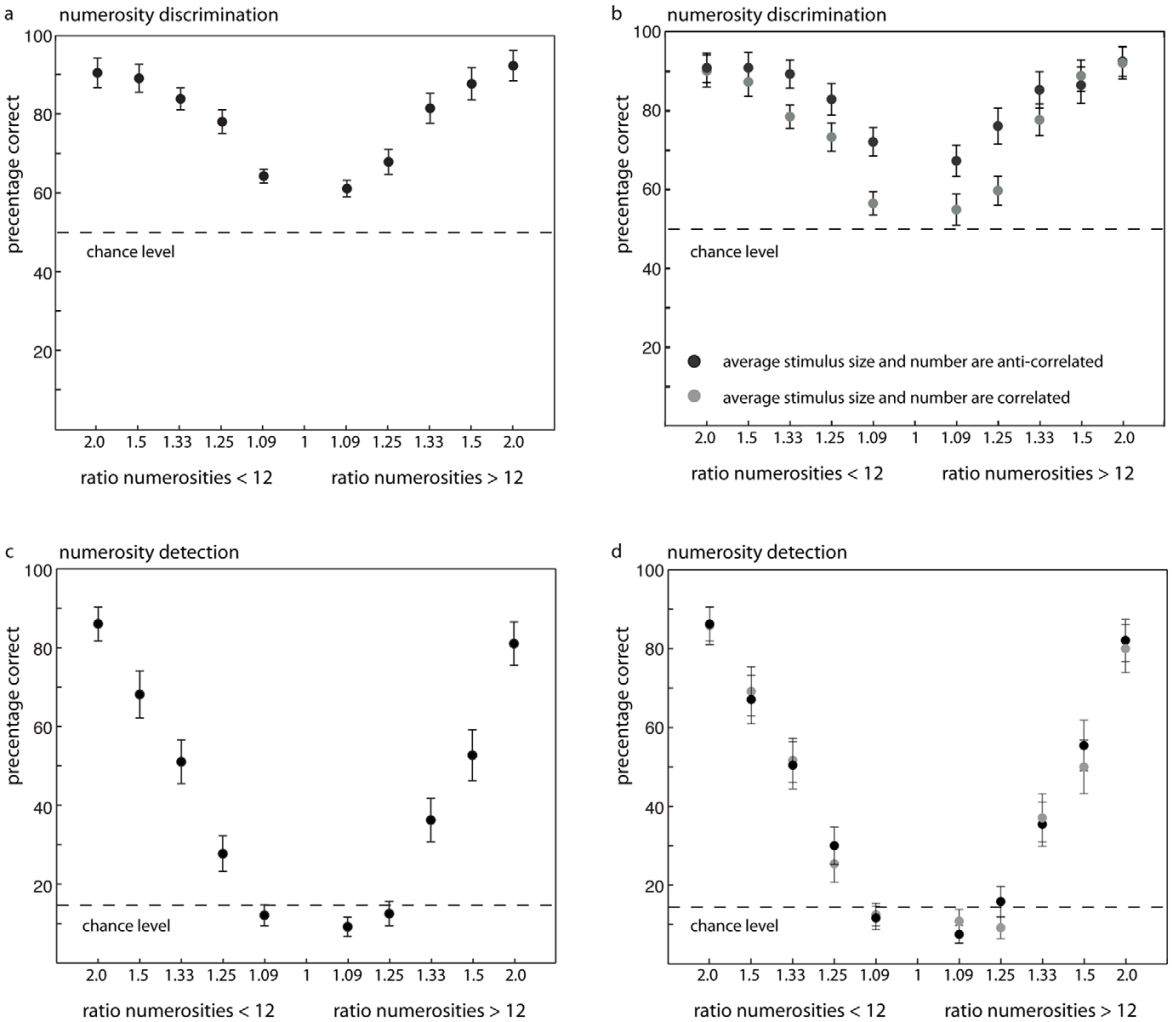
Performance on the discrimination (a, b) and detection task (c, d) as a function of ratio for both numerosities <12 and numerosities >12. The dashed line represents chance level, which was 50% for the discrimination task and 14% for the detection task. The left panels (a, c) show averaged data, the right panels (b, d) show performance for the trials where numerosity and visual cues were anti-correlated (black dots) and correlated (grey dots). The anti-correlated trials are the trials where each visual property of the more numerous dot-array was smaller. The correlated trials are the trials where the more numerous dot-array consisted of larger visual properties. The result that subjects performed better when number and visual cues were anti-correlated (black compared to grey dots) implicates that subjects more often identify the more numerous stimulus as being more numerous when it consists of smaller visual properties than the less numerous stimulus.

For the *discrimination task*, subjects performed significantly above chance level in all ratio conditions (all t's >5.205; all p's<0.001) (see Figure 2a). In contrast, performance on the *detection task* was only significantly above chance level for the seven largest ratio conditions (all t's >2.81; all p's<0.01) but not for the three smallest ratio conditions (ratio 1.09 (11 dots) [t(23) = -1.62, p = 0.12] / ratio 1.08 (13 dots) [t(23) = -2.53, p = 0.19] / ratio 1.17 (14 dots) [t(23) = -0.28, p = 0.78]) (see Figure 2c). These results implicate that the discrimination task and the detection task are not comparable in difficulty.

For the *discrimination task*, the results of the repeated measures ANOVA revealed a significant main effect for visual cue [F(1,23) = 21.97, p<0.001, η_p_^2^ = 0.49]: subjects performed better when visual cues and numerosity were anti-correlated (88% correct) than when they were correlated (78% correct) (see Figure 2b). Note that subjects perceived the dot array containing smaller dots as more numerous (better performance when number and visual cue were anti-correlated than correlated). Although contrary to the expectations, it is consistent with previous findings [19], [20], [21]. In addition, a main effect for stimulus size was present [F(1,23) = 7.29, p = 0.013, η_p_^2^ = 0.24], as subjects performed better in trials where smaller (compared to larger) numerosities had to be compared to twelve (see Figure 2b). We also obtained a significant main effect for ratio [F(4,92) = 181.99, p<0.001, η_p_^2^ = 0.89] suggesting better performance with increasing ratio. The interaction between visual cue and ratio also reached significance [F(4,92) = 10.28, p<0.001, η_p_^2^ = 0.31]. Post-hoc pair-wise comparisons showed that subjects performed significantly worse in the trials with correlated visual-cues than those with anti-correlated visual-cues (p's <0.001), in the three smallest ratio conditions. Apparently when task difficulty increased, the tendency of the subjects to rely on visual cues to judge numerosity also increased. The interaction between stimulus size and ratio also reached significance [F(4,92) = 5.77, p<0.001, η_p_^2^ = 0.20]. In the two smallest ratio conditions subjects performed better (both post-hoc pair-wise comparisons: p's <0.02) when twelve dots had to be compared to a number smaller than twelve than to a number larger than twelve. Stimulus size did not interact with visual cues [F(1,23) = 0.12, p = 0.734, η_p_^2^ = 0.05] and the interaction between ratio, visual cue and stimulus size did not reach significance either [F(4,92) = 1.69, p = 0.158, η_p_^2^ = 0.07]. We also looked at the “same trials”, the trials where both dot-arrays consisted of 12 dots. These trials also revealed that subjects indicated the array consisting of smaller dots, smaller aggregate surface and smaller contour length more often as the array containing more dots [t(23) = 2.55, p = 0.02]. Thus, subjects were influenced by visual cues when the difference between the two numerosities presented was small. Moreover, stimulus size affected judgment in the smallest ratio conditions, showing better performance for numerosities smaller than twelve.

For the *detection task* no significant main effect for visual cues was obtained [F(1,23) = 3.95, p = 0.059, η_p_^2^ = 0.15]; there was no consistent difference in the pattern of performance for trials where numerosity and visual cues were correlated or anti-correlated (see Figure 2d). Instead, there was a significant main effect of stimulus size [F(1,23) = 12.67, p = 0.002, η_p_^2^ = 0.36] suggesting that subjects performed better when the deviant numerosity was smaller than baseline (47% correct) compared to larger than baseline (38% correct). In addition, a significant main effect of ratio [F(4,92) = 167.04, p<0.001, η_p_^2^ = 0.88] was obtained. Performance increased when the relative difference between the numerosity deviant and baseline value increased. Stimulus size and ratio also interacted [F(4,92) = 2.59, p = 0.042, η_p_^2^ = 0.10]. Post-hoc pair-wise comparisons revealed better performance for smaller than for larger numerosities in some cases (ratio 1.2, 1.33 and 1.5; p's <0.044 but not ratio 1.09 and 2.0 p's >0.086). Visual cue and ratio interacted as well [F(4,92) = 2.89, p = 0.026, η_p_^2^ = 0.11]. Post-hoc pair-wise comparisons showed a significant effect for ratio 1.5 only (p = 0.01; for the remaining ratios p's >0.059). It can therefore be concluded that there is no consistent pattern in the reliance on visual cues when judging numerosity in the detection task. Neither the interaction between visual cue and stimulus size [F(1,23) = 0.31, p = 0.586, η_p_^2^ = 0.01] nor the three-way interaction reached significance [F(4,92) = 1.3, p = 0.274, η_p_^2^ = 0.05]. Thus, subjects detected the deviant numerosities more frequently in the large-ratio conditions and when numerosities were smaller than twelve. The duration of the detection task was approximately four times longer than the discrimination task. To investigate whether task duration could have affected the results we compared performance on the first, second, third and the fourth 25% of the trials. The results showed that performance did not improve (due to the establishment of a more reliable presentation of the baseline over the course of the task) or deteriorate (due to fatigue) [F(3,69) = 1.78, p = 0.159, η_p_^2^ = 0.07].

We also investigated the relation between performances on both tasks. The results revealed a significant correlation between performance on the discrimination and the detection task (p<0.02, R^2^ = 0.23). This is not surprising as this relation most likely reflects a general factor such as differential intelligence, use of attentional resources or motivation of the subjects.

The results of the regression analyses for the *discrimination task* showed that mathematical abilities significantly explained the variance in performance (F(1,4) = 4.04, p = 0.016, R^2^ = 0.459). More specific: performance on the discrimination task was only significantly explained by addition (t = 2.98, p = 0.008), and not subtraction (t = 0.42, p = 0.678), division (t = -0.88, p = 0.392) or multiplication (t = 0.62, p = 0.542). In contrast, for the *detection task*, mathematical abilities did not significantly explain the variance in performance (F(1,4) = 1.86, p = 0.16, R^2^ = 0.282). This outcome again exposes a difference between both measures of numerosity acuity.

Taken together, these results show that performance in the detection task was worse compared to performance for discrimination. We hypothesized that this difference in task difficulty could relate to the uncertainty induced by the lower chance level in detection (14%) compared to discrimination (50%). In experiment 2 we will investigate the effect of chance level on performance in both tasks.

## Experiment 2

To investigate the role of chance level, we equalized chance level for both tasks. To this end, the detection task was changed into a two alternative forced choice task, in which half of the trials contained two (consecutively presented) dot-arrays representing the same number of dots whereas the other half of the trials contained two dot-arrays representing a different number of dots. Subjects were asked to decide whether the two dot patterns represented an equal or a different number of dots (same-different task). If subjects perform this task equally well as they do in the discrimination task, the difference in chance level most likely caused the difference in performance obtained in experiment 1. However, if subjects perform worse on the same-different compared to the discrimination task, the difference in performance should be ascribed to the task (or task instruction) itself. This would be consistent with recent findings showing that discrimination tasks do not measure numerical abilities but reflect a decision process whereas same-different studies do measure numerical abilities [16], [17].

### Methods

Fifteen subjects participated in this study (aged between 25 and 34 years; 10 female, 5 male). All subjects were native Dutch speakers and had normal or corrected-to-normal vision. Written informed consent was obtained according to the Declaration of Helsinki and as approved by the local Ethical Committee. Contrary to experiment 1, the detection task was changed into a two alternative forced choice task to have a chance level of 50%. Now subjects had to decide whether two consecutively presented dot arrays represented the same or a different number of dots (same-different task). In the analyses of same-different studies, same trials are often excluded [16], [17]. We chose a different approach, since a response bias towards responding ‘different’ would artificially improve performance. Another measure that was taken to address this problem was that subjects were told in advance that chance level was 50% in both tasks. Furthermore, the stimuli were presented in blocks, each containing a single ratio condition. For the same-different task, each block now contained 12 same and 12 different trials. In this manner we could assign the same trials to a specific ratio condition. The different ratio blocks were fully randomized between participants. To keep the two tasks comparable, the stimuli in the discrimination task were also grouped per ratio condition and presented in fully randomized order. For both tasks the analyses were identical to those used in experiment 1.

### Results

**Figure 3:**
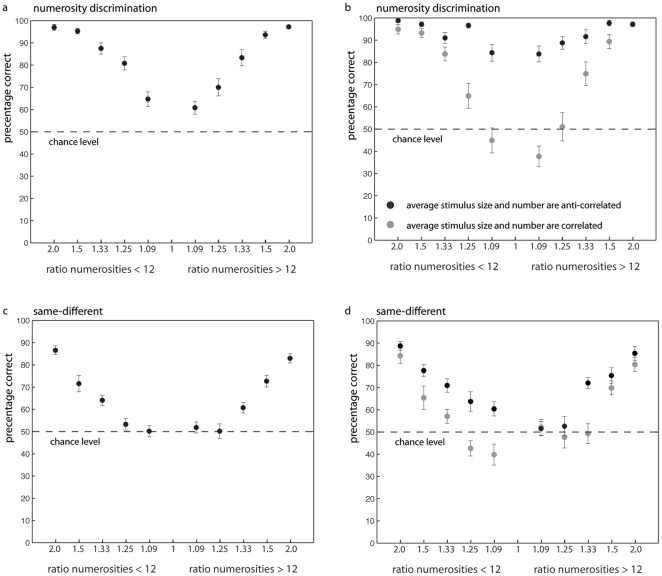
Performance on the discrimination (a, b) and same-different task (c, d) as a function of ratio for both numerosities <12 and numerosities >12. The dashed line represents chance level, which was 50% for both tasks. The left panels (a, c) show averaged data, the right panels (b, d) show performance for the trials where numerosity and visual cues were anti-correlated (black dots) and correlated (grey dots). The anti-correlated trials are the trials where each visual property of the more numerous dot-array was smaller. The correlated trials are the trials where the more numerous dot-array consisted of larger visual properties. The result that subjects performed better when number and visual cues were anti-correlated (black compared to grey dots) implicates that subjects more often identify the more numerous stimulus as being more numerous when it consists of smaller visual properties than the less numerous stimulus.

Similar as for experiment 1, for the *discrimination task*, subjects performed significantly above chance level in all ratio conditions (all t's >3.7; all p's <0.003) (see Figure 3a) whereas performance on the *same-different task* was only significantly above chance level for the six largest ratio conditions (all t's >4.58; all p's <0.001) and not the four smallest ratio conditions (ratio 1.2 (10 dots) [t(14) = 1.29, p = 0.22] / ratio 1.09 (11 dots) [t(14) = 0.11, p = 0.91] / ratio 1.08 (13 dots) [t(14) = 0.79, p = 0.44] / ratio 1.17 (14 dots) [t(14) = 0.08, p = 0.93]) (see Figure 3c). As the same trials are generally not included in the analyses, we also investigated performance for the different trials only. The results revealed that inclusion of the same trials did not alter the results. Subjects still performed at chance for the four smallest ratio conditions (all p's >0.26). Together, the results show that chance level cannot explain the differences in performance in the detection and discrimination task of experiment 1.

The results of the repeated measures ANOVA revealed, for the *discrimination task*, a significant main effect for visual cues [F(1,14) = 69.62, p<0.001, η_p_^2^ = 0.83]: subjects performed better in the number and visual cue anti-correlated (93% correct) compared to correlated trials (73% correct) (see Figure 3b). In addition, the main effect for stimulus size was not significant [F(1,14) = 3.66, p = 0.076, η_p_^2^ = 0.21]; subjects did not perform better in trials where smaller (compared to larger) numerosities had to be compared to twelve. Performance improved with increasing ratio as indicated by the significant main effect for ratio [F(4,56) = 82.93, p<0.001, η_p_^2^ = 0.86]. A significant interaction between visual cues and ratio was also present [F(4,56) = 28.72, p<0.001, η_p_^2^ = 0.67]. Apparently, subjects' reliance on visual cues increased when the difference in numerosity decreased. The two-way interaction between stimulus size and ratio [F(4,56) = 2.19, p = 0.081, η_p_^2^ = 0.14] as well as stimulus size and visual cues showed a trend towards significance [F(1,14) = 3.99, p = 0.066, η_p_^2^ = 0.22]; the three-way interaction between ratio, visual cue and stimulus size did not reach significance [F(4,56) = 1.19, p = 0.33, η_p_^2^ = 0.08]. Thus, subjects' performance was influenced by visual cues. This reliance on visual cues increased when the difference between the numbers to be compared decreased.

For the *same-different task* a significant main effect for visual cues was obtained [F(1,14) = 27.92, p<0.001, η_p_^2^ = 0.66]; subjects more often correctly identified the anti-correlated trials (70%) than the correlated trials (59%) as being same or different in number (see Figure 3d). No significant main effect of stimulus size was present [F(1,14) = 1.3, p = 0.27, η_p_^2^ = 0.09] suggesting that subjects' responses were not influenced by the absolute (numerical) size of the stimuli. In addition, a significant main effect for ratio [F(4,56) = 65.79, p<0.001, η_p_^2^ = 0.83] was obtained: performance increased when the relative difference between the two stimuli increased. Visual cue and ratio interacted [F(4,92) = 2.48, p<0.05, η_p_^2^ = 0.15]: subjects' reliance on visual cues increased when the relative numerical distance decreased. Neither the interaction between visual cue and stimulus size [F(1,14) = 2.78, p = 0.12, η_p_^2^ = 0.17] nor the interaction between stimulus size and ratio reached significance [F(4,56) = 0.72, p = 0.58, η_p_^2^ = 0.05]. However, the three-way interaction between visual cues, stimulus size and ratio did reach significance [F(4,56) = 3.3, p = 0.02, η_p_^2^ = 0.19].

We also compared overall performance between both tasks. Similar as in experiment 1, the results revealed a significant correlation between performance on the discrimination and the same-different task (p<0.01, R^2^ = 0.61). This is not surprising as this relation in overall performance between both tasks most likely reflects a general factor such as differential intelligence, use of attentional resources or motivation of the subjects.

As was the case for the discrimination task, subjects' performance on the same-different task depended on the relative numerical distance between the two stimuli. Performance was also influenced by the visual cues present in the stimuli and this influence increased with decreasing distance between the two numbers presented. This again was similar to the discrimination experiment. However, the overall performance on the same-different task was again much worse than performance on the discrimination task. Similar to performance in the detection task of experiment 1, subjects only performed above chance when the to be compared numbers differed with a ratio of 1.33 or more. In contrast, subjects reliably indicated which number was larger in the discrimination task when the numbers differed with a ratio of 1.08 or more. Since chance level was equal in both tasks of experiment 2, this implies that the difference in performance between the same-different and discrimination tasks can only be attributed to the task (instruction).

## Discussion

The development of the approximate number system (ANS), the system that is suggested to relate to mathematical abilities [1], [10], has been frequently investigated in infants using detection and in children and adults using discrimination tasks. As results from infant and child or adult studies are often directly compared [7], it is essential to know whether both tasks indeed tap into the same system and if so, at the same level. In the current study we therefore tested whether both paradigms are in fact comparable. In the first experiment, we employed both a detection and discrimination task in which the stimulus conditions, such as visual cues and ratio between the numerosities to be compared were identical. In addition we tested the relation between performance in both experiments to performance on (simple) mathematics tests. In the second experiment we used a modified version of experiment 1 to investigate whether differences in performance between the two tasks of experiment 1 could be attributed to chance level.

The stimuli to be compared in the discrimination and detection task (experiment 1) were identical and therefore should have been of equal difficulty. Nevertheless, subjects performed much worse in the detection task, where performance was above chance level for ratio differences of 1.33 and larger only. In contrast, subjects performed already above chance for ratio 1.08 in the discrimination task. This much better performance for discrimination compared to detection implicates a considerable *difference* in task difficulty. The task that is generally used for infants appears to be more difficult than the one used for children and adults. Interestingly, this implies that, either the performance of infants has been underestimated or that of adults overestimated. This has implications for current ideas about the mechanisms underlying the development of the ANS. It has been questioned, for instance, whether the development of ANS acuity depends on the acquisition of language [7]. The gradual increase in the development of ANS acuity, as described in current models, has been interpreted as evidence against a role for language acquisition. However, our data shows that this gradual change could be coincidental: due to the differences in task difficulty between infants and children or adults, the acuity of infants could be much higher, or that of children and adults lower. In our *detection task* adults could differentiate numerosities differing with a ratio of 1.33 and larger, which fits nicely to the data of nine-month-old infants that can dissociate dot patterns differing with a ratio of 1.5 [4]. Thus, when adult detection performance is used as measure, numerosity detection abilities appear to slightly increase between infancy and adulthood. Nevertheless it can be questioned to what extend passive viewing and active comparison can be compared. Future studies on adults, employing the same task as well as neuroimaging measures would be useful to further increase the comparability between results from infant and child or adult studies.

One factor that might explain the difference in performance between detection and discrimination is the difference in chance level in both paradigms (14% in the detection task versus 50% in the discrimination task). The difference in chance level could have led to an increased level of uncertainty in the detection task. However, in experiment 2, where chance level was equal in both tasks, subjects still performed worse when they had to decide whether two stimuli were equal in numerical size or not, compared to deciding which of the stimuli was numerically larger. Apparently, chance level was not the factor determining the difference in performance, but rather the task instruction, and with that the decision process. This notion is in agreement with the model of Piazza et al. ([18], see supplemental material) where it was shown that making a decision on whether two stimuli are numerically the same is more difficult than deciding which of two stimuli is the numerically larger (or smaller). Whereas discrimination and same-different tasks appear to measure different decision processes, the opposite seems apparent for the detection and the same-different task. Both these tasks led to comparable performance. This similarity in performance implicates that both are likely to gauge similar decision processes.

Not only overall performance, but also the subjects' strategy, i.e. the *reliance on visual cues* (be it implicitly or explicitly) differed between the detection and discrimination task. In the *discrimination task*, especially when task difficulty increased (ratio decreased), subjects were more likely to rely on visual cues. Subjects more frequently indicated the number and visual cues anti-correlated stimuli (larger number consists of smaller dots, smaller aggregate surface and is less dense, and vice versa) as being larger in number. That is, 12 dots were more often judged as being more than 12 when the stimulus was made up of smaller dots, smaller aggregate surface and was less dense, while they were judged more often as being less than 12 when larger dots, larger aggregate surface and higher densities were used. This was contrary to our expectations but coincides with the few studies investigating this relationship [19], [20], [21]. This reliance on visual cues in a numerosity discrimination task has been demonstrated before and appears to be more pronounced in younger children [22]. In contrast, in the *detection task*, subjects did not consistently rely on the visual properties of the stimuli when judging numerosity. Interestingly, in our second experiment we replicated the results for the discrimination task but now also obtained an effect of visual cues for the same-different task. Why subjects relied on the visual cues in a more consistent manner in the same-different compared to the detection task can only be speculated upon. It could be related to the difference in chance level as this was the only factor that differed between the two tasks. In the present experiments, the reliance on visual cues could not affect overall performance, as number and visual cues were correlated in one half, and anti-correlated in the other half of the trials. Indeed, overall performance was similar for the detection and the same-different task. Nevertheless, for neuroimaging results of number comparison studies the reliance on visual cues when judging number remains problematic. The increased reliance on the visual cues with decreasing number distance makes both processes correlated. As both number and different visual properties of the stimuli (e.g. luminance and physical size) have been shown to activate IPS regions [23], [24], [25], activation related to visual processes and number processes cannot be disentangled (for a more extensive discussion about visual confounds see [15]).

Our results show that the same different task might be a viable candidate to study the development of numerical abilities from infancy to adulthood. The results obtained using this task are more compatible with the results derived from infant studies and thus allows comparison of performance across age groups. However, Cantlon et al. [26] showed that stimulus heterogeneity (size, color, shape) influences performance on same-different but not discrimination tasks in children of 3 to 4 years of age. Similarly, Rousselle et al. [27] showed that heterogeneity does not influence performance on a comparison task in 3-year olds. Instead controlling for all visual cues resulted in random responses. Apparently both same-different and comparison tasks can be problematic for measuring number processes in 3-year olds. The fact that 3-year olds cannot differentiate numerosities irrespective of the task at hand is intriguing, especially when considering the results of studies that show that infants can make same-different judgments even when the stimuli are heterogeneous [14], [28]. The main difference between the infant studies and those of the 3-year olds is that the former relies on implicit and the latter on explicit measures. Around the age of 3, children learn to understand the counting principles, that is, the explicit rules about number. Possibly, saying two sets of items are the same (in number) while they look different or saying that something is larger (in number) while it is smaller (in size) can be confusing when a certain level of abstraction is not yet met. Although no clear relationship was found between performance on a non-symbolic comparison task and mastery of counting principles [29], the difference in explicit and implicit measures could be a reliable explanation. Using neuroimaging techniques, which allow implicit measuring of numerosity abilities, in concurrence with the behavioural task might overcome this problem.

The ANS has recently been suggested the precursor for mathematics achievement at a later age. Our results show that performance on the *discrimination task* can indeed be explained by performance on the mathematics tests, although addition appears the only significant contributing factor. In contrast, no relation was apparent between performance on the *detection task* and mathematical abilities. The discrepancy among these results might suggest that performance on the discrimination task is a more sensitive predictor of mathematical abilities than performance on the detection task. Consequently, the ANS acuity derived from discrimination tasks might be a better measure for predicting future mathematical abilities. However, the discrepancy in results could also suggest that the relation between ANS acuity and mathematics achievement is not as evident as sometimes suggested. Inconsistencies between studies investigating the relationship between non-symbolic number tasks and (future) mathematical abilities have been reported [1], [10], [11], [12], [30].

To conclude, the present results indicate that future studies trying to model the development of ANS acuity should only include results from studies that use the same paradigm across developmental stages. Since performance on the same-different task is similar to that in the detection task, the same-different task would be a viable alternative to discrimination tasks administered to children and adults. This would ensure better compatibility with the detection type tasks used with infants. In addition, as mentioned above, previous studies have shown same-different tasks to more purely measure numerosity representation, in contradistinction to discrimination tasks.
